# Are accreditation standards fit for measurement uncertainty in clinical laboratories? An analysis of Saudi Arabia’s accreditation

**DOI:** 10.3389/fmed.2026.1813990

**Published:** 2026-07-17

**Authors:** Majdi A. Aljohani

**Affiliations:** Department of Medical Laboratory Technology, Faculty of Applied Medical Sciences, University of Tabuk, Tabuk, Saudi Arabia

**Keywords:** accreditation, analytical performance specifications, biological variation, CBAHI, clinical chemistry, ISO 15189, measurement uncertainty

## Abstract

**Background:**

Measurement uncertainty (MU) is a fundamental aspect of laboratory quality management and is required by many international accreditation standards, including ISO 15189 and CAP ISO 15189. Nevertheless, its regulatory status under Saudi Arabia’s national accreditation system (CBAHI) remains unclear. This study aimed to examine the extent to which CBAHI accreditation standards implement the MU and their potential effect on patient safety.

**Methods:**

This study used a structured standards gap analysis to investigate MU coverage in CBAHI clinical laboratory standards. We analyzed seven CBAHI laboratory standards that are applicable to MU. This was against eight MU-related analytical domains derived from ISO 15189:2022. Alignment was scored on a 3-level scale (0 = no explicit requirement, 1 = partial alignment, 2 = full alignment). Then, we developed comparative benchmarking that compares MU expectations across ISO 15189:2022, the CAP 15189 accreditation program, and CLSI guidance. A biological variation framework from the EFLM Biological Variation Database was used to derive analytical performance specifications (APS) for MU. We focused only on high-impact analytes responsible for diagnosing high-impact diseases.

**Results:**

CBAHI standards exhibited moderate to high alignment with ISO 15189:2022 in conventional analytical quality domains, such as internal quality control, proficiency testing, calibration, and integration within the quality management system. Critically, MU lacked explicit coverage across all seven CBAHI standards (score 0). In contrast, many international guidance and standards consider MU a fundamental, mandatory requirement, including ISO 15189:2022, CAP ISO 15189, and CLSI guidance. The biological-variation-based APS for 10 clinical chemistry analytes were frequently close to commonly used diagnostic thresholds, underscoring the potential for theoretical misclassification when MU is unquantified.

**Conclusion:**

The current CBAHI clinical laboratory accreditation framework does not incorporate explicit requirements for MU estimation, performance limits, or clinical reporting. It is crucial to enhance national standards by incorporating MU-focused clauses, providing technical assistance, and integrating them into regular quality management processes. These measures are important for aligning CBAHI with global best practices and reducing the theoretical risk of diagnostic misclassification at critical decision points.

## 1 Introduction

**Figure 1:**
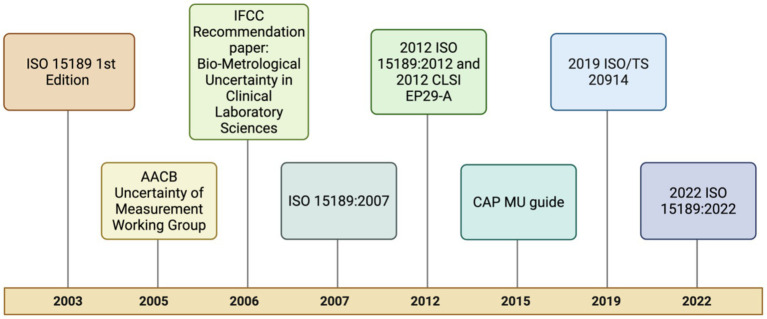
Timeline of measurement uncertainty (MU) guidance in clinical laboratories.

Laboratory testing plays a central role in diagnostic accuracy, clinical decision-making, and patient outcomes. In the clinical laboratory, the safety and reliability of reported results are influenced by pre-analytical, analytical, and post-analytical factors (1). One of the most critical components of a reliable interpretation of laboratory medicine results is an adequate assessment of measurement uncertainty (MU). It is a non-negative parameter indicating the range of values that could logically be attributed to the measured quantity (2). The inherent variability of the measurement process is frequently obscured by the accuracy suggested by numerical test results. Over the years, many accreditation and guidance standards have made MU a mandatory component of clinical laboratory quality systems (Figure 1). A recent observational study conducted in the US estimated that diagnostic errors occur in approximately 5% of adult emergency visits, including those caused by MU. The rate of misdiagnosis reported in many studies has not changed significantly over the years despite advances in discovery (3). The diagnostic error rates may be sustained by the failure to incorporate MU into the interpretation of results, as MU quantifies the reliability of reported values.

Building on this, MU can define the limit of doubt that allows the identification of the difference between an accurate diagnostic and a clinical decision that may be adverse (4). Failure to consider MU when patient results are near critical decision thresholds or reference interval limits may result in a misdiagnosis (5). For example, to avoid misclassification around the diagnostic cutoff of 126 mg/dL, the German Diabetes Association advises that the minimal difference (MD) indicating MU should not exceed 12.6 mg/dL in fasting plasma glucose (6). Furthermore, the analytical imprecision of the troponin decision threshold led to the incorrect diagnosis of high-risk patients with true values above the 99th percentile as negative (7). Moreover, in a prospective economic analysis of febrile children, diagnostic uncertainty was demonstrated to have significant downstream cost implications (8). These findings highlight the direct relationship between MU and misdiagnosis of many diseases (9).

**Figure 2:**
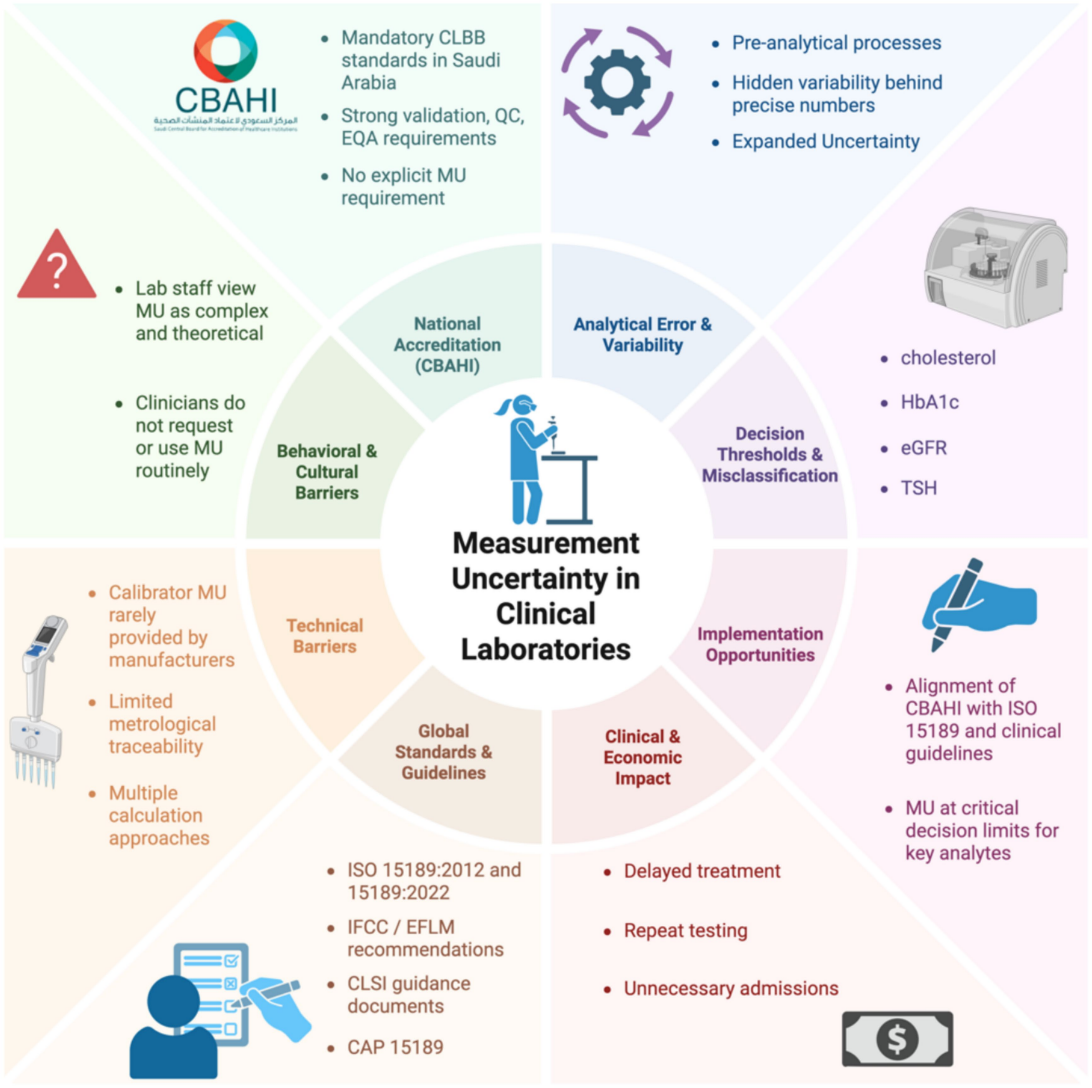
Conceptual framework summarizes determinants and consequences of measurement uncertainty in clinical laboratories.

At the methodological level, MU in clinical laboratories is usually estimated using metrological methods that incorporate data on traceability and imprecision (4). Building on these concepts, awareness of MU was further solidified in 2012, when ISO 15189:2012 mandated MU under Clause 5.5.1.4. As a result, there was a transition from the traditional emphasis on imprecision and bias to a comprehensive, patient-centered assessment of the accuracy of results. However, the implementation and harmonization still exhibit notable inconsistency more than a decade after the standard was adopted (10). Moreover, there is no standardized or widely accepted formula for MU estimation (2, 4). Medical laboratory personnel frequently view MU as theoretically accurate yet practically difficult (4, 10). Together, the most significant issue is that the majority of clinicians do not actively request these data (11). Along with these behavioral barriers are technical challenges because of the availability of metrological traceability (12, 13). As a result, the practical value of MU in improving clinical decision-making at the laboratory–clinician interface remains underutilized (Figure 2) (14).

In this context, clinical laboratories in Saudi Arabia are a relevant setting for investigating this implementation gap, particularly given the 2013 policy change that mandated CBAHI accreditation (15). It is the responsible authority that can issue accreditation certificates to all governmental and private healthcare institutions, including clinical laboratories (16). The CBAHI Clinical Laboratory and Blood Bank Standards (CLBB) explain governance and organizational structure requirements. Additionally, they mention the procedures that are essential for the successful completion of a laboratory quality management program. In general, these standards underscore the importance of ensuring that test results are clinically accurate, traceable, and reliable. Nevertheless, it is crucial to conduct a comprehensive examination of the CBAHI Clinical Laboratory Standards. Comparing their requirements with international standards is necessary to understand their alignment and practical impact in Saudi Arabian laboratories. To date, no study has systematically assessed the extent to which CBAHI requirements mitigate measurement uncertainty in Saudi clinical laboratories.

Within this accreditation framework, MU is a significant component of laboratory quality management systems (17). It is an important aspect of the effectiveness of an analysis and directly influences the reliability of patient results (18). Many studies have confirmed that medical laboratories may face consequences, including inaccurate results, if the MU is not taken into consideration (10, 19, 20). In clinical chemistry, recent research has demonstrated that patient findings that are near diagnostic thresholds are susceptible to misclassification when the MU exceeds Analytical performance specifications (APS). This can lead to inappropriate treatment and misdiagnosis (21, 22). Such misclassifications have been reported for several key analytes including lactate dehydrogenase, creatine kinase, and serum sodium. These findings emphasize that addressing the implementation gap is essential for patient safety.

To illustrate these concerns at the analyte level, we selected a set of high-impact measurands for analyte-specific investigation in order to demonstrate the clinical impact of MU at critical decision thresholds. This includes glucose, HbA1c, creatinine, total cholesterol, high-sensitivity cardiac troponin T, and TSH. These analytes were chosen because they provide a substantial basis for diagnostic or therapeutic decision-making at a clearly defined clinical threshold. Moreover, each analyte is provided with biological variation data and analytical performance specifications that are recommended by the guideline. The desired imprecision and total-error specifications for these measurands were obtained from biological-variation-based APS that were sourced from the EFLM Biological Variation Database and associated publications. These specifications enabled us to quantify the potential adverse effects and patient misclassification that could result from MU near critical thresholds.

It is unclear to what extent Saudi Arabia’s national accreditation standards (CBAHI) explicitly mandate MU estimation, despite the prominent role of MU in international accreditation frameworks. The present investigation systematically assesses how MU is addressed in the context of CBAHI accreditation. It aims to clarify current expectations for MU by analyzing the CBAHI criteria along with essential international frameworks, thereby emphasizing the regulatory–practice gap. It also intends to establish objectives for integrating MU into clinical decision-making and laboratory quality management processes of Saudi Arabia. This study clarifies the operationalization of MU requirements by linking CBAHI requirements to ISO 15189:2022 clauses and BV-based APS at the analyte level. This is the first systematic mapping of a national accreditation standard to ISO 15189:2022 at the analyte level, in conjunction with BV-based APS.

## 2 Materials and methods

### 2.1 Study design and conceptual framework

**Figure 3:**
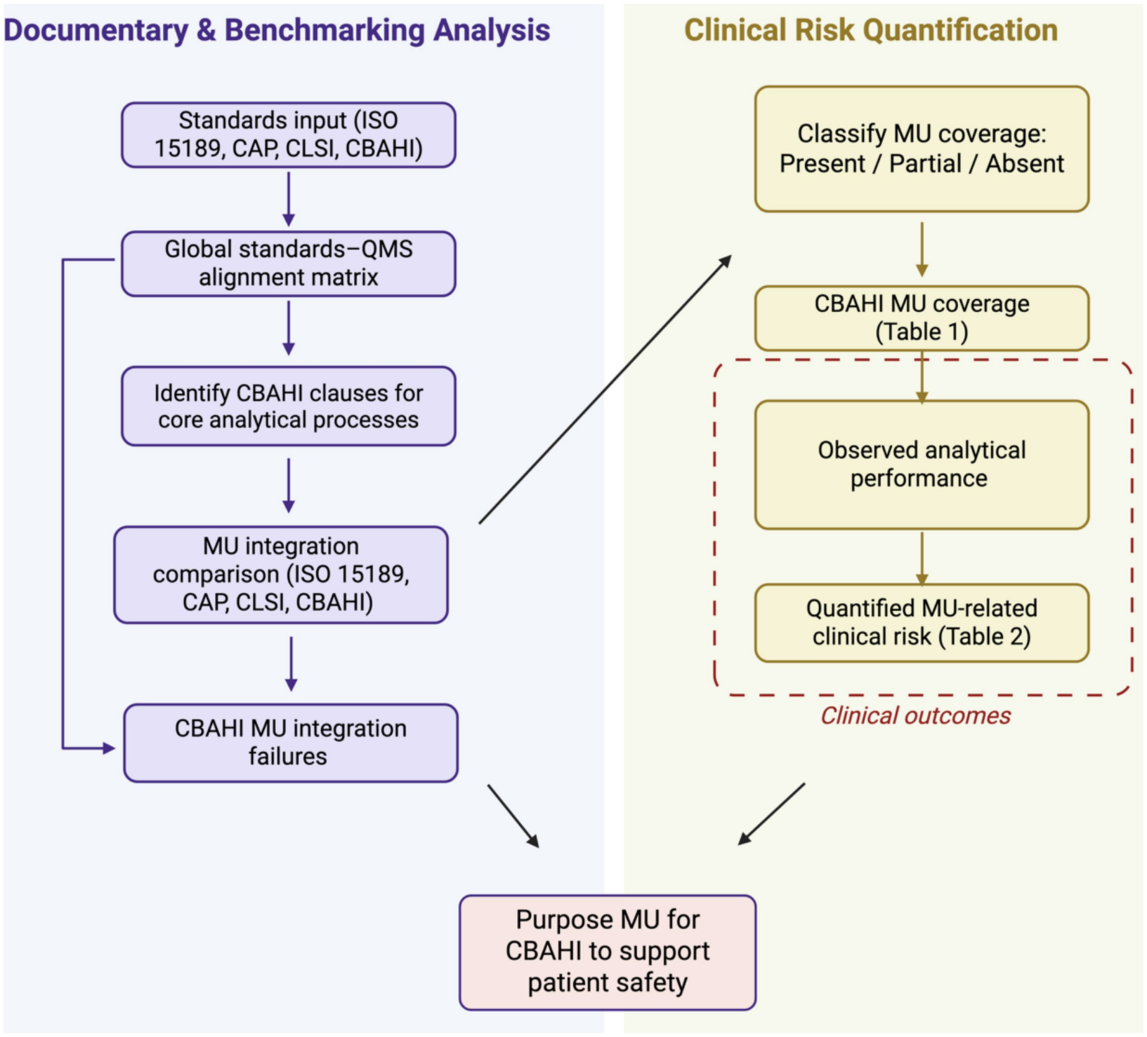
Two-phase methodological framework for evaluating CBAHI accreditation standards.

This study used a structured standards gap analysis to assess the extent to which the CBAHI clinical laboratory standards are implemented by the MU (Figure 3). Initially, we identified seven CBAHI laboratory standards applicable to MU and its use in clinical decision-making. These standards include method validation, quality control, method–instrument correlation, reference intervals and cutoff values, proficiency testing, calibration and standardization of critical equipment, and validation of critical equipment. For these standards, we generated a mapping matrix against ISO 15189:2022, identifying clauses that either establish MU requirements or regulate analytical processes that provide the primary inputs for MU estimation and application. The ISO clauses included the following: measurement uncertainty, validation and verification of examination procedures, internal quality control, external quality assessment/proficiency testing, calibration and metrological traceability, establishment and verification of reference intervals, management of critical values, and integration within the quality-management system. We compared MU-related content with international reference standards (CAP and CLSI) to examine the potential implications for patient risk.

The seven CBAHI standards were selected based on whether the standard directly or indirectly contributes to MU estimation, reporting, or clinical application. Moreover, the standard corresponds conceptually to at least one ISO 15189:2022 clause that addresses MU or MU-relevant analytical processes. Standards unrelated to quantitative analysis, including personnel competency, facility safety, and specimen transport, were excluded as MU is not applicable. The eight ISO 15189:2022 analytical domains were selected using parallel criteria: (i) the clause explicitly mandates MU estimation or reporting or (ii) the clause governs an analytical process including validation, calibration, quality control, proficiency testing that supplies primary data inputs for MU calculation. Furthermore, clauses limited to pre-analytical or post-analytical processes unrelated to MU were excluded. This dual selection framework ensures that the gap analysis captures both direct MU requirements and the upstream analytical processes that generate MU inputs. Moreover, it minimizes selection bias through explicit, pre-defined inclusion rules.

### 2.2 Ethical considerations

Structured standards gap analysis was implemented in this study without personal or identifiable data. The analysis was conducted exclusively through the methodical evaluation of national standards frameworks and accessible accreditation documents.

### 2.3 Data sources and collection

Two primary sources were used to obtain the data for the standards-based analysis: (1) the CBAHI Clinical Laboratory Standards, 1st edition, 2015 and (2) international MU-related reference documents including ISO 15189:2022. To establish international best practices for MU-specific accreditation, guidance documents from CAP, and CLSI were consulted. The EFLM Biological Variation Database was used to derive the coefficients of within-subject biological variation (CVI) in MU performance (Table 1). This approach was used to ensure alignment with current evidence-based recommendations.

**Table 1:** Analytes that have performance targets derived from biological variance, no defined CBAHI APS/MU requirements, and related clinical decision risk.

Analyte	B (CVI %)*	Desirable imprecision (APS %)**	CBAHI Explicit APS/MU requirement	Clinical decision limit	Potential of unquantified MU
Albumin	2.5	1.3	No	3.5–5.0 g/dL (adult reference interval)	Affects CKD staging, dialysis prognosis (26)
Glucose	4.7	2.4	No	126 mg/dL (diabetes diagnosis)	Impacts the diagnosis of DM (6)
Creatinine	4.4	2.2	No	eGFR 60 mL/min/1.73 m^2^ (CKD G3a threshold)	Over-diagnosis of some kidney disease (27)
Sodium	0.6	0.3	No	135–145 mmol/L (adult reference interval)	Sodium MU commonly exceeds APS (Treatment Errors and Mortality Risk) (21)
Potassium	3.9	2.0	No	5.5 mmol/L (hyperkalemia)	Unnecessary treatment (28)
Calcium	1.8	0.9	No	8.5–10.5 mg/dL (total calcium reference interval)	Affect treatment decisions (29)
Cholesterol	5.3	2.7	No	200 mg/dL (risk threshold)	Affect treatment decisions (30)
HbA1c	1.7	0.9	No	6.5% (diabetes diagnosis)	Impacts the diagnosis of DM (14)
Troponin T	12.2	6.1	No	14 ng/L (99th percentile, Roche Elecsys hs-TnT)	Unnecessary invasive procedures and antithrombotic therapy (31)
TSH	17.8	8.9	No	0.4–4.0 mIU/L (adult reference interval)	Near these thresholds can be misclassified (32)

*EFLM data; **0.5 × CVI model.

### 2.4 Standards mapping and coding protocol

**Figure 4:**
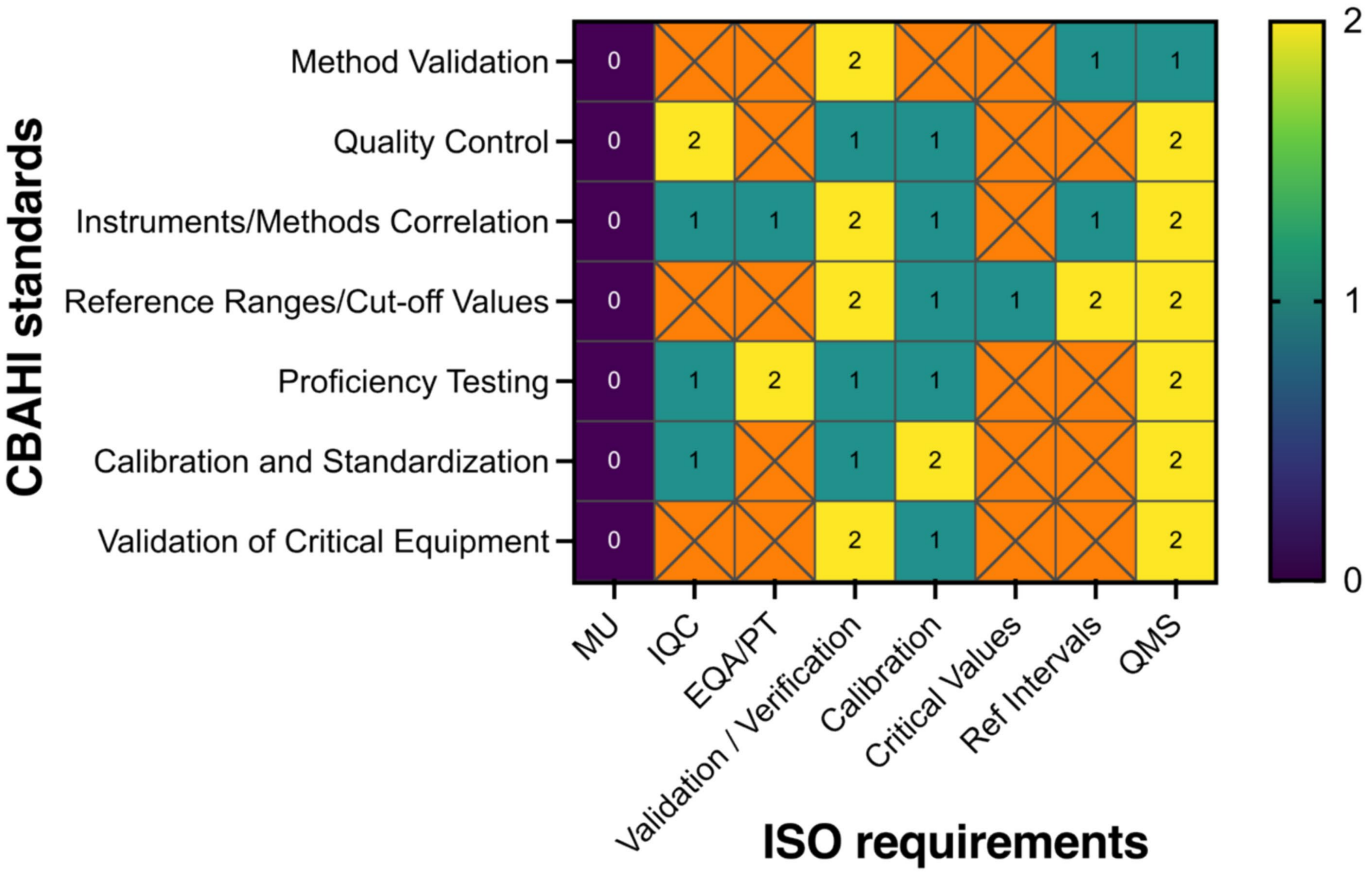
Heat map of alignment between CBAHI standards and ISO 15189:2022 analytical requirements. Tiles scored (0) no explicit requirement, (1) partial alignment, and (2) full alignment. Cross cells indicate non-applicable combinations (excluded from calculations).

The standards mapping was conducted in a systematic, ordered approach. This method ensured that the comparison between CBAHI requirements and international standards could be reproduced. Initially, the key clauses and associated content regarding MU were extracted from ISO 15189:2022. In parallel, the CBAHI Clinical Laboratory and Blood Bank Standards were reviewed line-by-line to identify conceptually corresponding requirements. The CBAHI standards were mapped against these domains and categorized into three alignment categories. Fully aligned, which indicates that CBAHI contains clauses that are conceptually equivalent to the ISO domain (score = 2). Partially aligned, which indicates CBAHI includes text that addresses part of the ISO requirement (score = 1). Finally, not explicit which indicates CBAHI has no clause that addresses the ISO domain content (score = 0). Not-applicable combinations were excluded from scoring and alignment calculations, and are denoted by a cross in Figure 4. This classification facilitated a systematic gap analysis between the requirements of local and international MU. To reduce subjective bias in alignment scoring, all seven CBAHI standards were independently reviewed and coded by two reviewers. Inter-rater agreement was assessed for 56 scoring decisions across 7 CBAHI standards and 8 ISO topic domains. The initial coding phase was conducted with reviewers unaware of each other’s scores, and all disagreements were resolved through consensus discussion. The two reviewers agreed on 47 of 56 scores (83.9% observed agreement). Cohen’s kappa (*κ*) was calculated across categories as 0.761 (95% CI: 0.62–0.90), indicating substantial agreement between reviewers.

### 2.5 MU international benchmarking

We developed a comparative benchmarking table that compares MU expectations across ISO 15189:2022, the College of American Pathologists (CAP) 15,189 accreditation program, and The Clinical & Laboratory Standards Institute (CLSI) guidance. The MU requirement status for each framework was categorized as either mandatory, recommended, or absent. Moreover, the MU estimation approach (e.g., bottom-up models and top-down models) was specified for each framework. Additionally, we determined whether numeric analytical performance specifications or other allowable limits were explicitly demanded as the benchmark against which MU should be assessed for each standard.

### 2.6 Integration with clinical performance data

A biological variation framework was used to derive analytical performance specifications (APS) for measurement uncertainty. The EFLM Biological Variation Database was used to obtain the coefficients of within-subject biological variation (CVI) for each analyte. We focused on high-impact analytes including glucose, HbA1c, creatinine, total cholesterol, high-sensitivity cardiac troponin T, and TSH. These analytes are responsible for the diagnosis of high-impact diseases including diabetes, chronic kidney disease, myocardial infarction, and thyroid dysfunction. Subsequently, standard EFLM formulations were used to determine the desired APS for each analyte in terms of imprecision and total measurement uncertainty. The most desirable performance specification tier was selected for all analytes, as it is the level of analytical performance that enables clinical decisions to be made with minimal additional risk beyond inherent biological variation. The minimum and optimum tiers were not used because the minimum tier allows for clinically significant misclassification rates, while the optimum tier is rarely achieved in real-world settings using routine laboratory methods. The purpose of this selection was to establish a relationship between MU and clinically established decision limits. These APS based on biological variation were subsequently contextualized in relation to the assay’s observed analytical imprecision and pertinent clinical decision limits for interpretation.

### 2.7 Data management and statistical analysis

All extracted standard clauses and alignment ratings were compiled into a structured spreadsheet matrix (Microsoft Excel), with separate worksheets for: (i) the CBAHI-ISO alignment matrix and justification notes, (ii) the initial scores from each reviewer, and (iii) the final consensus scores after discussion. The alignment between CBAHI and ISO 15189:2022 requirements was quantified using descriptive statistics. For each alignment category, the frequency and proportion of CBAHI standards were determined. As a benchmark for evaluation, desirable analytical performance specifications (APS) were derived from the biological variation of the clinical chemistry analyte panel. We then directly compared the total MU for each test with the desirable specifications to evaluate analytical performance. All statistical figures were generated using GraphPad Prism (GraphPad Software, San Diego, CA, USA).

## 3 Results

### 3.1 Alignment of CBAHI technical standards with ISO analytical requirements

The alignment matrix in Figure 4 compares seven CBAHI technical standards with eight fundamental ISO 15189:2022 analytical requirements. Alignment was quantified using a three-tier scoring system: (0) indicates no explicit CBAHI requirement, (1) indicates partial alignment, and (2) demonstrates full alignment. Crossed cells, representing comparisons outside the scope of a given standard, were designated as not applicable (N/A) and excluded from the quantitative analysis. In general, none of the CBAHI standards exhibited comprehensive coverage across all applicable ISO analytical domains. However, some domains exhibited significant alignments in specific areas. For example, quality management system (QMS) integration was consistent with most ISO clauses, each of which received a score of 2 in the QMS domain. The internal quality control (IQC) and calibration requirements were generally well represented, with 71 and 57% of standards achieving full alignment, respectively. Quality Control, Instruments/Methods Correlation, Proficiency Testing, and Calibration and Standardization exhibited moderate to high alignment with ISO 15189:2022. The core domain of the reference ranges/cutoff values standard demonstrated distinct strengths, including a strong linkage. Critically, the MU did not exhibit explicit coverage across all seven CBAHI standards. Inter-rater agreement for the alignment scores was substantial (*κ* = 0.76), supporting the reliability of the coding protocol.

### 3.2 International benchmarking: position of CBAHI

In international standards, MU is considered a fundamental, mandatory requirement. However, it is entirely absent from the current Saudi national accreditation (CBAHI). ISO 15189:2022 provides a comprehensive framework for MU, together with the technical specification ISO/TS 20914:2019. Laboratories are required to estimate MU for analytical data sources, such as method validation, internal quality control (IQC), proficiency testing (PT), and calibration records. Moreover, laboratories must compare the calculated uncertainty to pertinent analytical performance requirements (APS). Finally, ISO15189:2022 states that MU information must be easily accessible and provided to users upon request or during the examination of clinical data.

The College of American Pathologists (CAP) converts these guidelines into actionable practice by providing specialized guidance on MU. Moreover, it provides a checklist that is used to assess compliance. Laboratories are required to implement a documented MU, which is consistent with the principles delineated in the Guide to the Expression of Uncertainty in Measurement (GUM). Moreover, laboratories are required to evaluate their MU estimates against predefined performance benchmarks, including allowable total error limits or analytical performance specifications (APS). Finally, the program mandates that these estimates be easily accessible to authorized clinicians and other relevant parties to facilitate the development of clinical decisions.

Professional guideline documents from the Clinical and Laboratory Standards Institute (CLSI) provide a comprehensive approach to MU estimation. They published EP29 and others, which offer both top-down and bottom-up approaches for determining measurement uncertainty. These documents suggested including the establishment of MU as a critical component of laboratory best practice. The CBAHI CLBB standards do not include MU requirements. As a result, laboratories are not required to establish MU estimation for analytical performance, including those derived from biological variation. The benchmarking analysis in Table 2 reveals a discrepancy between CBAHI and other accreditation and guidance programs.

**Table 2:** Measurement uncertainty criteria are benchmarked internationally across accreditation and guidance systems.

Accreditation/standard	MU requirement status	Governing document/clause	MU estimation methodology specified?	Numeric APS/Allowable limits required?	MU reporting to clinicians required?	Geographic coverage
ISO 15189:2022 (accreditation standard)	Mandatory	Clause 7.3.4; ISO/TS 20914:2019	Yes (Bottom-up approach)	Yes	On request or when interpreting result	International
CAP ISO 15189 (accreditation standard)	Mandatory	MU Guide (2015); CAP checklist items	Yes (Bottom-up approach)	Yes	Available to authorized users	International
CLSI (guidance document)	Guidance	EP29-A (2012)	Yes (Both bottom-up and top-down approaches)	Yes	Recommended best practice	Global (guidance)
CBAHI (accreditation standard)	Absent	No clause in CLBB 2015 standards	Not specified	No	No mentioned	Saudi Arabia

### 3.3 Clinical context using biological variation data

Normally, it is recommended that analytical performance specifications (APS) be used to establish acceptable limits for MU for a variety of measurands, based on biological variation. As a result, we implemented a BV framework to contextualize MU at clinically significant decision thresholds for a panel of 10 analytes. The corresponding absolute allowable MU was determined by applying the BV-based desirable APS (%) to diagnostic decision limit for each analyte. Compared with the decision thresholds for glucose, creatinine, cholesterol, and HbA1c, these allowable uncertainty ranges were small (Table 1). For instance, the allowable MU for glucose (desirable APS = 2.4%, decision limit 126 mg/dL) is approximately ±3.0 mg/dL. This indicates that there is limited space for additional analytical variability before results near the diagnostic cutoff may be theoretically misclassified. None of the analytes had specific APS or MU requirements as specified in the CBAHI Clinical Laboratory and Blood Bank Standards. Furthermore, at the relevant clinical decision boundaries, CBAHI made no mention of performance targets based on biological variation. Table 1 lists the clinical decision limits used and summarises published qualitative descriptions of the potential impact of unquantified MU at those thresholds.

## 4 Discussion

This analysis shows that CBAHI accreditation remains limited, despite its structural robustness. It remains inconsistent with the most recent international standards for uncertainty quantification in clinical laboratories. In accordance with ISO 15189:2022, laboratories are required to estimate MU for relevant examinations using validation, IQC, EQA, and calibration data. Furthermore, the ISO 15189 program and CLSI guidance embed MU within routine quality activities using structured checklist items (23). This links the uncertainty estimation with other analytical performance specifications, such as the allowable total error (24). In contrast, the CBAHI CLBB standards incorporate the standard technical quality components, such as method validation, internal quality control, testing proficiency, and equipment maintenance. However, they do not include a clause that enforces MU estimation, defines acceptable uncertainty limits, or specifies when and how to provide this information to physicians.

The domain-level mapping underscores the issue of MU implementation at the national level, which results from the absence of MU requirements. Each of the primary CBAHI technical clauses examined in this study, including method validation, quality control, method correlation, reference ranges, proficiency testing, calibration, and equipment validation, was classified as non-explicit with respect to MU. At the same time, a significant number of these domains were completely compliant with the quality management standards of ISO. This indicates that the issue is not a deficiency in structural resilience, but rather the lack of a clear mandate to transform existing data streams into quantitative uncertainty estimates. This may affect the interpretation of those estimates in relation to clinical decision limits or analytical performance specifications. As a result, laboratories may never consider the uncertainty of their test results when answering specific clinical questions.

To underscore the clinical implications of this regulatory gap, we conducted an analyte-specific analysis using EFLM biological variation data. We focused on high-impact measurands including glucose, HbA1c, creatinine, cholesterol, troponin T, and TSH. The desirable BV-based APS often comes close to the diagnostic decision limits. This means a significant portion of typical diagnostic decision limits can be represented by the allowable MU. For example, even with low serum creatinine concentrations that correlate with GFR levels, small analytical errors have greater effects (25). Even a minor degree of MU can have a significant impact on how patients are categorized in relation to this decision limit. As a result, our BV-based modeling suggests that ignoring increases in MU could theoretically cause patients to be incorrectly classified above diagnostic thresholds for diabetes, chronic kidney disease, cardiovascular risk, myocardial infarction, or thyroid dysfunction. Nevertheless, Table 1 illustrates that CBAHI does not specify APS or MU expectations for any of these analytes, even though this remains a theoretical risk in the Saudi context. A comprehensive investigation is needed to directly assess the impact of MU on clinical decision-making at critical diagnostic thresholds in Saudi laboratories.

**Figure 5:**
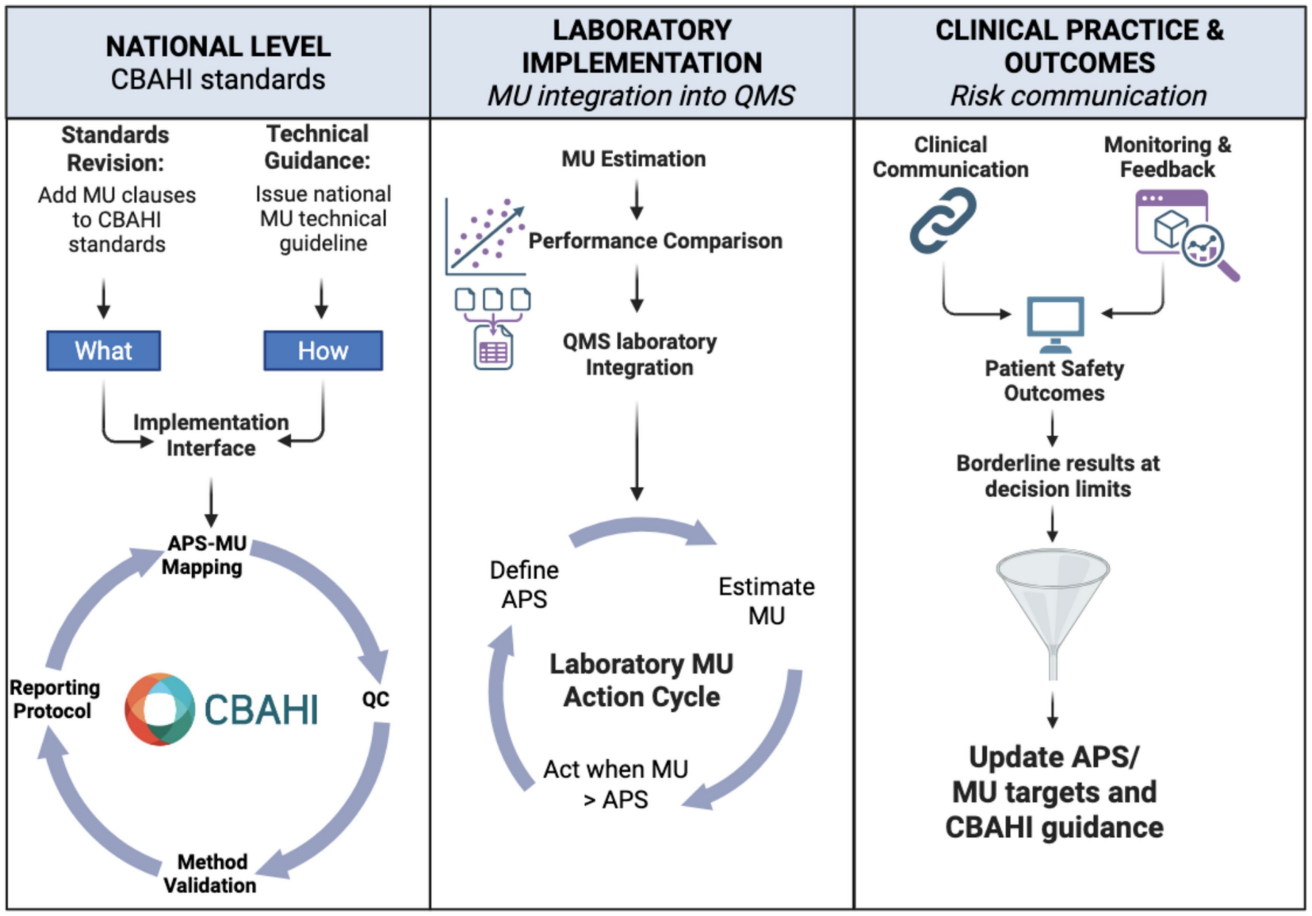
Proposed multi-level framework for integrating measurement uncertainty (MU) into Saudi laboratory practice.

To overcome this issue, we propose an implementation plan across three levels: national, laboratory, and clinical practice (Figure 5). At the national level, CBAHI has the capacity to modify its national standards to incorporate explicit MU clauses that are consistent with ISO 15189:2022 and other international standards. In addition, it has the potential to publish a national MU technical guideline that explains the process by which laboratories should estimate MU for different domains. Moreover, they can translate it into APS-MU requirements at each stage of the quality cycle. At the laboratory level, a regular MU action cycle can be implemented to incorporate MU estimation into the QMS, which can involve APS. At the clinical level, structured risk communication can be implemented to periodically revise the APS/MU targets and CBAHI guidance. This method would prioritize feedback loops from clinicians, patient safety outcomes, and outcomes that are unclear at decision limits.

## 5 Limitation

The analysis is document-based, which implies that any unpublished updates, informal guidance, or surveyor practices are not considered. We use EFLM CVI data and widely used decision limits to demonstrate the practical implications of unquantified MU at common diagnostic thresholds. The APS used in this analysis are derived exclusively from the biological variation model. This may not accurately represent all Saudi patient populations, as we did not measure actual MU or imprecision in Saudi laboratories.

## 6 Conclusion

For MU in clinical laboratories, the CBAHI accreditation framework does not yet meet the international standards. However, CBAHI demonstrates a comprehensive platform for traditional technical quality management, as illustrated in this analysis. There are no requirements in CBAHI standards that require a MU estimate, quantitative APS criteria, or a formal association between MU and clinical decision limitations. This contrasts with other accreditations and guidance that recommend or mandate the MU in clinical labs, including ISO 15189:2022, CAP ISO 15189 guidance, and CLSI documents. The omission is not only theoretical, as the analyte-level context of EFLM biological variation-derived APS illustrates in this analysis. The theoretical risk that Saudi laboratories may not systematically evaluate MU in relation to critical decision thresholds is indicated by the absence of explicit MU requirements in this document-based and modeled analysis. Consequently, future revisions of CBAHI standards are required and should include explicit MU requirements.

## Data Availability

The original contributions presented in the study are included in the article/>Supplementary material, further inquiries can be directed to the corresponding author/s.

## References

[1] Fenta DA Ali MM. Factors affecting quality of laboratory result during ordering, handling, and testing of the patient’s specimen at Hawassa University College of Medicine and Health Science Comprehensive Specialized Hospital. J Multidiscip Healthc. (2020) 13:809–21. doi: 10.2147/JMDH.S264671, 32922023 PMC7450655

[2] Kurtulmuş Y Tan R Yilmaz M. A practical approach example to measurement uncertainty. Biochem Med. (2022) 32:396–404. doi: 10.11613/BM.2022.030705, 35966261 PMC9344870

[3] Singh H Meyer AND Thomas EJ. The frequency of diagnostic errors in outpatient care: estimations from three large observational studies involving US adult populations. BMJ Qual Saf. (2014) 23:727–31. doi: 10.1136/bmjqs-2013-002627, 24742777 PMC4145460

[4] Milinković N Ignjatović S Šumarac Z Majkić-Singh N. Uncertainty of measurement in laboratory medicine. J Med Biochem. (2018) 37:279–88. doi: 10.2478/jomb-2018-0002, 30584397 PMC6298468

[5] Timbrell NE. The role and limitations of the reference interval within clinical chemistry and its reliability for disease detection. Br J Biomed Sci. (2024) 81. doi: 10.3389/bjbs.2024.12339, 38481978 PMC10932992

[6] Keutmann S Zylla S Dahl M Friedrich N Landgraf R Heinemann L . Measurement uncertainty impacts diagnosis of diabetes mellitus: reliable minimal difference of plasma glucose results. Diabetes Therapy. (2020) 11:293–303. doi: 10.1007/s13300-019-00740-w, 31845101 PMC6965559

[7] Lyon AW Kavsak PA Lyon OAS Worster A Lyon ME. Simulation models of misclassification error for single thresholds of high-sensitivity cardiac troponin I due to assay Bias and imprecision. Clin Chem. (2017) 63:585–92. doi: 10.1373/clinchem.2016.265058, 27974385

[8] Leigh S Grant A Murray N Faragher B Desai H Dolan S . The cost of diagnostic uncertainty: a prospective economic analysis of febrile children attending an NHS emergency department. BMC Med. (2019) 17:48. doi: 10.1186/s12916-019-1275-z, 30836976 PMC6402102

[9] Mills NL Lee KK McAllister DA Churchhouse AMD MacLeod M Stoddart M . Implications of lowering threshold of plasma troponin concentration in diagnosis of myocardial infarction: cohort study. BMJ. (2012) 344:e1533. doi: 10.1136/bmj.e1533, 22422871 PMC3307810

[10] Padoan A Antonelli G Aita A Sciacovelli L Plebani M. Issues and challenges in applicability of measurement uncertainty estimation in medical laboratories. J Lab Precis Med. (2017) 2:69. doi: 10.21037/jlpm.2017.08.10 28245184

[11] Plebani M Sciacovelli L Bernardi D Aita A Antonelli G Padoan A. What information on measurement uncertainty should be communicated to clinicians, and how? Clin Biochem. (2018) 57:18–22. doi: 10.1016/j.clinbiochem.2018.01.017, 29402416

[12] Beastall GH. Traceability in laboratory medicine: what is it and why is it important for patients? EJIFCC. (2018) 29:242–7. 30574032 PMC6295586

[13] Siekmann L. Requirements for reference (calibration) laboratories in laboratory medicine. Clin Biochem Rev. (2007) 28:149–54. 18392129 PMC2282407

[14] Galindo-Méndez M Sánchez-López A Cruz-Fuentes L. The estimation of uncertainty of measurement of glycated hemoglobin as an analytical performance specification and in the interpretation of its results. Clin Biochem. (2019) 63:92–6. doi: 10.1016/j.clinbiochem.2018.10.012, 30595159

[15] Almasabi M Thomas S. The impact of Saudi hospital accreditation on quality of care: a mixed methods study. Int J Health Plann Manag. (2017) 32:e261–78. doi: 10.1002/hpm.2373, 27435144

[16] D MA A FA S AA M ME M AH. Evolution of the accreditation program for healthcare organizations in KSA: from present to future. J Taibah Univ Med Sci. (2024) 19:130–2. 37964863 10.1016/j.jtumed.2023.10.002 PMC10641268

[17] Nellutla R Chowdary R. Point of view on measurement uncertainty in terms of quality management. J Medical Scientific Research. (2015) 3:198–202. doi: 10.17727/JMSR.2015/3-038

[18] Braga F Panteghini M. The utility of measurement uncertainty in medical laboratories. Clinical Chemistry Laboratory Medicine. (2020) 58:1407–13. doi: 10.1515/cclm-2019-1336, 32126011

[19] Akşit M Demirci F. Measurement uncertainty of HbA1c and glucose parameters, which are diabetes mellitus diagnostic tests. Anatolian J General Medical Research. (2024) 84–90:84–90. doi: 10.4274/anatoljmed.2024.97720

[20] Pecori Giraldi F Ambrogio AG. Variability in laboratory parameters used for management of Cushing’s syndrome. Endocrine. (2015) 50:580–9. doi: 10.1007/s12020-015-0676-9, 26160393 PMC4662716

[21] Wadhwa N Bhat K Kalsi M Sadhu T. Evaluation of measurement uncertainty in clinical chemistry and its comparison with analytical performance specifications. Cureus. (2025) 17:e79043. doi: 10.7759/cureus.79043, 40099095 PMC11912806

[22] Venturim JR Vieira LV. Estimates of measurement uncertainty associated with clinical laboratories and their implications for clinical practice. Revista Brasileira de Análises Clínicas. (2024) 56:242–52. doi: 10.21877/2448-3877.202400216.en

[23] Kallner Anders. Expression of Measurement Uncertainty in Laboratory Medicine: Approved Guideline. Clinical and Laboratory Standards Institute: International Federation of Clinical Chemistry and Laboratory Medicine, (2012).

[24] Haeckel R Wosniok W Gurr E Peil B. Permissible limits for uncertainty of measurement in laboratory medicine. Clinical Chemistry Laboratory Medicine. (2015) 53:1161–71. doi: 10.1515/cclm-2014-0874, 25720082

[25] Peake M Whiting M. Measurement of serum creatinine--current status and future goals. Clin Biochem Rev. (2006) 27:173–84. 17581641 PMC1784008

[26] Infusino I Panteghini M. Serum albumin: accuracy and clinical use. Clin Chim Acta. (2013) 419:15–8. doi: 10.1016/j.cca.2013.01.005, 23348571

[27] Badrick T Turner P. The uncertainty of the eGFR. Indian J Clin Biochem. (2013) 28:242–7. doi: 10.1007/s12291-012-0280-1, 24426218 PMC3689324

[28] Zhou R Qin Y Padoan A Sciacovelli L Aita A Wang Q . Different approaches for estimating measurement uncertainty: an effective tool for improving interpretation of results. Clin Chim Acta. (2020) 503:223–7. doi: 10.1016/j.cca.2019.11.011, 31733194

[29] Sandberg S Coskun A Carobene A Fernandez-Calle P Diaz-Garzon J Bartlett WA . Analytical performance specifications based on biological variation data – considerations, strengths and limitations. Clinical Chemistry Laboratory Medicine. (2024) 62:1483–9. doi: 10.1515/cclm-2024-0108, 38501489

[30] ÇAT A UÇAR KT. Calculation of measurement uncertainty of 20 clinical chemistry Analytes according to the practical ISO approach. Acibadem Universitesi Saglik Bilimleri Dergisi. (2023) 14. doi: 10.31067/acusaglik.1174521

[31] The importance of evaluating measurement uncertainty for troponin I. Annals Clinical Analytical Medicine. (2019) 10. doi: 10.4328/JCAM.5835, 31733194

[32] Bhattacharya A Mukherjee K Dasgupta A Mallick D Mukhopadhyay M. Finding order in measuring disorders: measurement uncertainty of thyroid function tests at a tertiary care hospital in eastern India. Asian J Med Sci. (2025) 16:31–7. doi: 10.71152/ajms.v16i6.4530

